# Editorial: Green nanomaterials: prospective biotechnological applications

**DOI:** 10.3389/fmicb.2023.1280398

**Published:** 2023-10-13

**Authors:** Amr Fouda, Arnab Bhowmik, Saad El-Din Hassan, Mohamed Hijri

**Affiliations:** ^1^Department of Botany and Microbiology, Faculty of Science, Al-Azhar University, Nasr City, Cairo, Egypt; ^2^Department of Natural Resources and Environmental Design, North Carolina Agricultural and Technical State University, Greensboro, NC, United States; ^3^Institut de Recherche en Biologie Végétale (IRBV), Département de Sciences Biologiques, Universitéde Montréal, Montréal, QC, Canada; ^4^African Genome Center, Mohammed VI Polytechnic University (UM6P), Ben Guerir, Morocco

**Keywords:** nanomaterials, nanoparticles, microbial extracts, bacteria, fungi, antimicobacterial, metabolite

In recent years, the intersection of nanotechnology and sustainable practices has given rise to a fascinating and rapidly evolving field known as “Green Nanomaterials”. These innovative materials are derived from renewable resources and produced with environmentally friendly processes, offering immense potential for a diverse range of biotechnological applications. Growing concerns about the ecological impact of traditional materials and the escalating demand for sustainable solutions have directed attention toward green nanomaterials, exploring their unique attributes and potential benefits, along with exciting prospects in the field of biotechnology (Bandara et al., 2020). Green nanomaterials are intriguing due to their eco-friendly production methods, reduced toxicity, resource efficiency, and versatility across industries. They offer health benefits in medicine, address environmental issues, enhance agriculture, advance technology, reduce waste, and align with ethical considerations. Overall, they provide solutions to diverse global challenges while minimizing harm to the environment and human health. Bacteria, fungi, and plants have become the driving force behind the creation of nanomaterials with unique structures, sizes, compositions, and properties (Wang et al., 2016; Akther et al., 2019). Nanotechnology intersects with nature, wherein plants and microorganisms play an instrumental role in nanomaterials production as well as in their biotechnological applications.

Among these applications, Khodeer et al. used *Phragmanthera austroarabica* extract, along with gallic acid, is used in the synthesis of silver nanoparticles (AgNPs), ensuring they maintain a uniform shape and consistent size distribution. This dual role of the extract and gallic acid serves as both reducing and capping agents. Furthermore, the inclusion of gallic acid extract on the nanoparticle surface enhances the surface-to-volume ratio and, in turn, augments its pharmacodynamic efficacy. Khodeer et al. found evidence that AgNPs may enhance the antidiabetic effects of *P. austroarabica* extract and gallic acid. These formulations have a positive effect on various parameters, including body weight, lipid profile, insulin resistance, hyperinsulinemia, and liver tissue structure and function. Additionaly, they have been shown to increase the activity of pathogenic bacteria. The extracts-based on AgNPs showed significant antibacterial efficacy, suggesting that they could be used as disinfectants in healthcare environments. Balan et al. (2016) reported that AgNPs' antidiabetic impact is linked to their ability to inhibit digestive enzymes, such as α-amylase and α-glucosidase.

Thymol—a volatile monoterpenoid phenol—possesses antibacterial and antioxidant properties, and is found in medicinal plant essential oils (Singh et al., 2017). Chitosan nanoparticles loaded with thymol have been studied extensively with respect to their efficacy against foodborne-disease - causing microorganisms. In particular, Sreelatha et al. found that thymol-loaded chitosan nanoparticles (TCNPs) were able to inhibit *Xanthomonas campestris pv campestris*—the bacterium that causes black rot in brassica crops. However, the exact molecular mechanism by which *X. campestris pv campestris* reacts to TCNPs remains largely unknown. To uncover this, Sreelatha et al. employed physiological, spectroscopic, and untargeted metabolomics methods. The results showed that *X. campestris pv campestris* cells exposed to sub-lethal TCNP concentrations displayed altered cell proliferation and membrane damage, accompanied by changes in membrane potential. In addition, the intracellular metabolite profiles of TCNP-treated *X. campestris pv campestris* cells revealed changes in amino acids, lipids, nucleotides, fatty acids, and antioxidant metabolites. It is postulated that reactive oxygen species (ROS)-induced damage of the cell membrane and oxidative stress will cause changes in metabolism. This pathway would enable TCNPs to enter the cells, where they can interact with macromolecules like proteins and nucleic acids, leading to further ROS production and ultimately cell death. This knowledge could help to develop nanoparticle-based agrochemicals as a sustainable alternative to conventional pesticide.

Modern medicine, veterinary care, food production, and agriculture are challenging due to microbial diseases, which have become increasingly resistant to existing antimicrobial agents (Hashempour-Baltork et al., 2019; Mahdi et al., 2022). Antibiotic resistance is caused by the overuse and misuse of antibiotics in agriculture, healthcare, and the environment (Larsson and Flach, 2022; Mahdi et al., 2022). Biofilms, complex multicellular formations, are responsible for persistent infections by many dangerous microorganisms. Their self-synthesized structure—consisting of a hydrated organic matrix of exopolysaccharides, proteins, and nucleic acids—provides a robust protective environment for microbes (Olivares et al., 2020). Trzcińska-Wencel et al. successfully produced effective AgNPs using *Fusarium culmorum* strain JTW1. A range of characterization methods revealed the properties of the AgNPs, such as their small size, spherical shape, stability, crystalline nature, and biomolecule encapsulation. Nanoparticle size, shape, stability, and capping molecules can greatly influence the effectiveness of AgNP (Li et al., 2012). Studies by Trzcińska-Wencel et al. have shown that AgNPs are effective against human and plant bacterial infections. In particular, AgNPs improved the antibacterial efficacy of streptomycin against Gram-negative bacteria, ampicillin against *Pseudomonas aeruginosa*, and ampicillin and kanamycin against Gram-positive bacteria. AgNPs were also able to inhibit the formation of biofilm and biofilm-produced hydrolytic enzymes. These studies demonstrate AgNPs' exceptional ability to combat bacterial biofilms. These studies emphasize the exceptional ability of AgNPs to combat bacterial biofilms, making fungi-synthesized silver nanoparticles a viable solution for pathogen-related issues in agriculture and the food industry.

Recent studies have demonstrated a strong correlation–between secondary bacterial infections and COVID-19 mortality (Jose and Desai, 2020). *Pseudomonas aeruginosa* and methicillin-resistant *Staphylococcus aureus* (MRSA) are among the principal causative factors in COVID-19-related bacterial infections (Luyt et al., 2020; Bassetti et al., 2022). Therefore, Hawsawi et al. sought to investigated whether biosynthesized silver nanoparticles from strawberry leaves (*Fragaria ananassa* L.) could prevent pathogenic microorganisms from flourishing in sputum samples from COVID-19 patients. The noteworthy stability and lack of chemical catalysts during the synthesis process contribute to the environmentally friendly nature of the synthesized AgNPs. The results suggest that environmentally freindly production of FA-AgNPs provides an inexpensive option that could help impede the growth of bacteria extracted from COVID-19 patients, emphasizing their potential use in combating bacterial infections associated with the disease.

Microorganisms are recognized for their capacity to mineralize metal ions; nevertheless, it is unclear how they contribute to the mineralization of calcium carbonate. Microbial activity is a common natural process that produces calcium carbonate. It can be detected in a variety of microorganisms that perform metabolic processes such as denitrification, photosynthesis, and organic acid oxidation (Li et al., 2018; An et al., 2021; Lin et al., 2021). As a consequence, microbially induced calcium carbonate precipitation (MICP) has become a popular technological tool with a variety of applications such as soil enhancement, restoration of building materials, preservation of historical stone monuments, and environmental remediation (Lin et al., 2021). Li and Li evaluated MICP using *Saccharomyces cerevisiae* under various culture conditions. Transcriptome analysis revealed that organic acid oxidation seemed to enhance the yeast's respiratory processes, suggesting that *S. cerevisiae* contributes to calcium carbonate formation by producing an alkaline environment through the metabolic oxidation of organic acids. This understanding of the role of *S. cerevisiae* in the biomineralization process provides valuable insight.

In this ever-evolving landscape of innovation, the applications of green-created nanomaterials continue to progress. Green-created nanomaterials, have diverse future applications. They can enhance clean energy generation, aid in environmental cleanup, improve agriculture with eco-friendly pesticides and fertilizers, advance biomedicine through precise drug delivery and diagnostics, enable environmental monitoring, contribute to waste management and sustainable construction. Research into safety, environmental impact, and scalability is crucial for responsible adoption. These applications have the potential to transform industries, improve lives, and expand our understanding of what can be achieved with the intersection of biology and nanotechnology as both scientific research and technological progress keep moving forward.

## Author contributions

AF: Writing—review and editing. AB: Writing—review and editing. SH: Writing—original draft. MH: Writing—review and editing.
